# Weight‐loss interventions for improving emotional eating among adults with high body mass index: A systematic review with meta‐analysis and meta‐regression

**DOI:** 10.1002/erv.2906

**Published:** 2022-04-23

**Authors:** Han Shi Jocelyn Chew, Siew Tiang Lau, Ying Lau

**Affiliations:** ^1^ Alice Lee Centre for Nursing Studies Yong Loo Lin School of Medicine National University of Singapore Singapore Singapore

**Keywords:** behaviour, BMI, emotional eating, obesity, weight

## Abstract

**Objectives:**

To evaluate the effectiveness of weight‐loss interventions on emotional eating among adults with high body mass index (BMI).

**Methods:**

A systematic review, meta‐analysis and meta‐regression were performed on randomized controlled trials published from inception until 19 March 2021.

**Results:**

Thirty‐one studies were included, representing 1203 participants with mean ages ranging from 21.8 to 57.3 years old and BMI 27.2–43.5 kg/m^2^. We found small‐to‐medium interventional effects on emotional eating (*n* = 18; Hedges' *g* = 0.22; *p* = 0.01, *I* ^2^ = 61.7%), uncontrolled eating (*n* = 16; Hedges' *g* = 0.46; *p* < 0.001, *I* ^2^ = 71.6%) and cognitive restraint (*n* = 18; Hedges' *g* = 0.42; *p* < 0.001, *I* ^2^ = 75.8%). Small‐to‐medium interventional effects were only found for emotional eating (*n* = 8; Hedges' *g* = 0.45; *p* = 0.02, *I* ^2^ = 74.3%) 3‐month post‐intervention, and on BMI (*n* = 4; Hedges' *g* = 0.43; *p* < 0.05, *I* ^2^ = 33.4%) and weight (*n* = 6; Hedges' *g* = 0.36; *p* < 0.01, *I* ^2^ < 10.4%) 12‐month post‐intervention. Age, male proportion, baseline BMI, attrition rate and intervention length were not significant moderators of the heterogeneity between studies.

**Conclusion:**

Interventions improved emotional eating and weight loss along a year‐long trajectory.

## Highlights

- Weight‐loss interventions such as Cognitive Behaviour Therapy (CBT), diet and exercise, and mindfulness are effective in improving emotional eating, uncontrolled eating/external eating and cognitive restraint/restrained eating.
- Purely mindfulness‐based interventions showed a higher interventional effect size over a combination of CBT and mindfulness, CBT and diet and/or exercise.
- Small‐to‐medium interventional effect size on body mass index and weight was only observed 12‐month post‐intervention.

**Abbreviations**
ACT: acceptance and commitment therapy
BMI: body mass index
CBT: cognitive behaviour therapy
CINAHL: Cumulative Index to Nursing and Allied Health Literature
COVID‐19: coronavirus disease 2019
CNKI: China National Knowledge Infrastructure
DB: dialectical behaviour therapy
DEBQ: Dutch Eating Behaviour Questionnaire
EES: Emotional Eating Scale
MBI: mindfulness‐based interventions
PRISMA: Preferred Reporting Items for Systematic Reviews and Meta‐Analysis
RoB: Risk of Bias
SD: standard deviation
TFEQ: Three‐Factor Eating Questionnaire

## 1 INTRODUCTION

Overweight and obesity affects approximately 39% of the world's adult population (World Health Organization, 2020) and is known to increase one's risk of cardiometabolic diseases (Kivimäki et al., 2017), musculoskeletal disorders (Paulis et al., 2014), cancers (Steele et al., 2017) and infectious diseases (e.g., COVID‐19; Hamer et al., 2020). While typical weight management programs behaviour have been effective in promoting weight loss through caloric restrictions and increased physical activity (Gudzune et al., 2015), participants of such programs were shown to regain more than 80% of the weight lost within 5 years (Anderson et al., 2001). Such weight cycling has been associated with covert behavioural factors such as emotional eating (Braden et al., 2016; Chew et al., 2022), a behaviour that is commonly unaddressed in conventional weight‐loss programmes.

Emotional eating refers to the behaviour of eating in response to certain emotional triggers (especially negative emotions and stress) instead of our innate biological hunger (van Strien, 2018). More than half of the adults with obesity have been found to display characteristics of emotional eating (Péneau et al., 2013; Wong et al., 2020), increasing one's tendency to display dysfunctional eating behaviours such as binge‐eating and disinhibited/unrestrained eating (Escandón‐Nagel et al., 2018; Wiedemann et al., 2018). Such eating behaviours have in turn been associated with depression, weight gain, weight‐loss failure and weight regain (Braden et al., 2016; Risica et al., 2021). While there is no specific definition for what constitutes an ‘emotional eating intervention’, some weight‐loss interventions including physical activity, stress reduction, mindfulness‐based interventions (MBI), Acceptance and Commitment Therapy (ACT), Cognitive Behaviour Therapy (CBT) and Dialectical Behaviour Therapy (DBT; Lawlor et al., 2020; Frayn et al., 2018; Frayn & Knäuper, 2018). These interventions are mostly adapted from existing psychotherapeutic techniques to improve emotional regulation (Bilici et al., 2020; Michopoulos et al., 2015). Common instruments used to measure emotional eating includes the Three‐Factor Eating Questionnaire (TFEQ), Dutch Eating Behaviour Questionnaire (DEBQ), and the Emotional Eating Scale (EES; Frayn & Knäuper, 2018). However, the effectiveness of such interventions specifically among the population in need—adults with high body mass index (BMI; ≥25 kg/m^2^)—and the underlying mechanism by which they do so remains unclear.

Several systematic reviews have shown elusive findings on the effectiveness of such interventions on weight loss (Carriére et al., 2018; Katterman et al., 2014; Yu et al., 2020). For example, one systematic review reported that mindfulness meditation resulted in a medium‐to‐large effect on decreasing binge‐eating tendencies (Katterman et al., 2014). However, while two of the five included studies found significant reductions in emotional eating, only three of 10 studies found significant interventional effects on weight loss (BMI/weight; Katterman et al., 2014). Moreover, conclusions were reached based on the proportion of studies that reported significant interventional effects instead of a pooled effect size, which is a more rigorous way of evaluating the evidence (Haidich, 2010). On the other hand, another meta‐analysis reported that MBIs had a large effect on emotional dysfunctional eating habits (*n* = 10) such as emotional eating and a moderate effect on weight loss (*n* = 16; Carrière et al., 2018). However, this study had high heterogeneity (*I* ^2^ = 74.45–88.73) possibly due to the inclusion of studies targeted at both the general public and those with overweight/obesity. Dysfunctional eating habits namely emotional eating, binge eating and restrained eating, were also aggregated in the same meta‐analysis which could have contributed to the high heterogeneity (Carrière et al., 2018). A systematic review on the effectiveness of ACT on weight also showed insufficient evidence to support the use of ACT for overweight/obesity due to mixed findings from heterogeneous studies (Öst, 2014). Another study reviewed the effectiveness of ACT on overweight/obesity but merely listed the effectiveness on weight‐related outcomes such as emotional eating, value‐focussed behaviours, weight management, psychological flexibility, body satisfaction and quality of life (Yıldız, 2020). No meta‐analyses were conducted possibly due to the sheer number of and high heterogeneity between the studies. Moreover, the sustainability of such intervention effects was rarely examined. Although one review assessed the long‐term effectiveness of MBT on various outcomes, various follow‐up periods were pooled within a single meta‐analysis (Carrière et al., 2018). This could have reduced the accuracy of findings as the interventional effects could fluctuate with time.

Due to the shortcomings of previous systematic reviews, it is difficult to determine the effectiveness and hence the applicability of weight‐loss interventions on emotional eating and weight loss. To our best knowledge, there is no systematic review that comprehensively evaluates the evidence of weight‐loss interventions on emotional eating and weight loss specifically in adults with high BMI. Therefore, we aimed to conduct effect size analyses to evaluate the effectiveness of various weight‐loss interventions on emotional eating and weight loss among this population with the following objectives:

- To evaluate the effectiveness of different weight‐loss interventions on emotional eating and weight loss.
- To explore the potential covariates that impact the effect size of health outcomes.

## 2 METHODS

This study is reported according to the Preferred Reporting Items for Systematic Reviews and Meta‐Analysis (PRISMA; Moher et al., 2009; Supp. 1) and registered with the PROSPERO (Reference number: CRD42021251841).

### 2.1 Eligibility criteria

The inclusion criteria were developed based on the population, intervention, comparison, outcome and study design (PICOS) framework.

P: Community‐dwelling adults who were overweight or obese. We excluded articles that recruited predominantly participants with preexisting physical (e.g. diabetes mellitus) or mental illnesses (e.g. depression). Participants with eating disorders were included and analysed as subgroups.

I: Interventions targeted at reducing emotional eating. We excluded articles that focussed on the effects of surgical interventions, drug therapy or solely diet and exercise without counselling components targeted at reducing emotional eating.

C: Usual care or no intervention.

O: Emotional eating and/or weight loss. We excluded articles that did not assess emotional eating as an outcome.

S: Randomized controlled trials.

Articles that were not in English or Mandarin were removed.

### 2.2 Information sources and search terms

A search on PubMed and Cochrane library was first conducted to prevent a duplicated study on this topic. Once we had confirmed that no similar study could be found, eight electronic databases were searched from inception until 19 March 2021—CINAHL, Embase, PsycINFO, PubMed, MEDLINE, Scopus, The Cochrane Library, Web of science. To enhance the comprehensiveness of our search, we also searched for grey and Chinese literature on OpenGrey, the first 10 pages of Google scholar and CNKI. Additional articles were retrieved by a manual search of the reference lists of the included articles.

Search terms used were ‘emotional eating’, intervention*, trial*, program*, therapy, strateg*, ‘weight loss’, ‘weight reduction’, BMI, overweight, obes* and ‘high BMI’. Details on the search strings corresponding to each database are shown in Supp. 2.

### 2.3 Study selection

Articles were selected according to the eligibility criteria by the first author and reviewed by the second and third authors. Discrepancies were discussed as a group and the original authors of the articles were contacted to obtain missing data for effect size computation.

### 2.4 Data collection

Data extraction was performed by the first author using an excel spreadsheet with the following headers: Author, year, country of origin, type of publication, sample size, mean age, the proportion of males, baseline BMI, participant characteristics, attrition rate, weight measure, intervention, control condition, interventionist, intervention length, session duration, number of sessions per week, mode of delivery, individual or group, emotional eating measure, intervention and control group mean, standard deviation (SD), and sample size for emotional eating, binge eating and weight/BMI (post‐intervention and follow‐up).

### 2.5 Risk of bias in individual studies

The Cochrane Collaboration's Risk of Bias (RoB) tool was used to assess each article's methodological quality (Higgins et al., 2019). Each article was assessed independently by two authors (LST, HSJC) and discrepancies were resolved through discussion with the third reviewer when required. Each study was given a rating of low, unclear or high RoB according to each domain.

### 2.6 Synthesis of results

The Comprehensive Meta‐Analysis software (version 3, Biostat) was used to conduct the meta‐analyses (Bornstein et al., 2005). *Z*‐statistics at a significance level of *p* < 0.05 was used to analyse the overall effect. Hedges' *g* was adopted because it provides an accurate estimation of the corrected effect size for a small sample size. The effect size was interpreted as small (0.2), medium (0.5), large (0.8) and very large (1.2; Hedges & Olkin, 2014).

We conducted our meta‐analyses according to the three subfactors presented within TFEQ (i.e., emotional eating, uncontrolled eating and cognitive restraint) and DBEQ (i.e. emotional eating, external eating and restrained eating) given their popularity and similarity in the included studies (see Section 3.1. study characteristics). Therefore, effect sizes were pooled for the three subscales, binge eating, weight in terms of BMI and weight in terms of kilogram (kg) using random‐effects models for meta‐analyses.

I^2^ was classified as unimportant (40%), moderate (30%–60%), substantial (50%–90%) and considerable (75%–100%) heterogeneity (Higgins et al., 2019). Egger's test was used to assess for publication bias and presented using funnel plots.

### 2.7 Additional analyses

Meta‐regression analysis was conducted to explain whether the heterogeneity between trials could be attributed to covariates (Borenstein et al., 2021). Covariates considered were mean age, percentage of male participants, baseline BMI, attrition rate and intervention length (weeks). Subgroup analyses were also performed to compare the effects among the various region, eating patterns, types of intervention and control conditions on emotional eating, uncontrolled eating, cognitive restraint and BMI. The predefined subgroups included the region of study (country of origin was categorized into World Health Organization [WHO] regions), eating pattern (dysfunctional/normal eating), intervention type and control condition.

## 3 RESULTS

**FIGURE 1:**
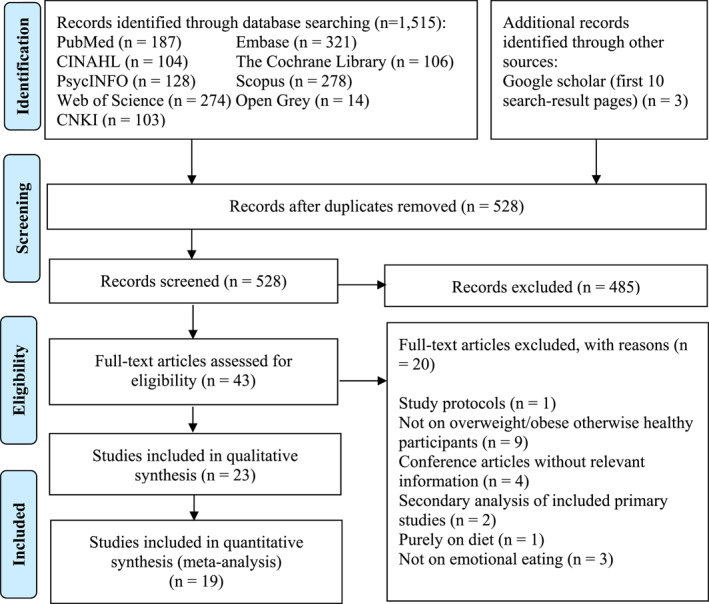
Flow diagram illustrating search strategy

A total of 1518 articles were originally retrieved. After removing duplicate articles, 528 articles were screened using their titles and abstracts of which 43 articles were screened for full texts. One additional article was included from reference list searching. We excluded 20 articles with reasons shown in Figure 1, resulting in a total of 23 RCTs included in this review. As eight studies comprised of more than one intervention (Cesa et al., 2013; Czepczor‐Bernat et al., 2020; Jarvela‐Reijonen et al., 2018; Kristeller et al., 2014; Kullgren et al., 2013; Manzoni et al., 2016; Mason et al., 2019; Stapleton et al., 2020), the 23 articles were analysed as 31 studies.

### 3.1 Study characteristics

The included studies were published from 2010 to 2021, representing a total of 1203 participants with mean ages ranging from 21.8 to 57.3 years old (Afari et al., 2019; Cesa et al., 2013; Czepczor‐Bernat et al., 2020; Forman et al., 2013; Gade et al., 2013; Goldbacher et al., 2016; Hjelmesæth et al., 2019; Jarvela‐Reijonen et al., 2018; Kim et al., 2020; Kristeller et al., 2014; Kullgren et al., 2013; Manzoni et al., 2016; Mason et al., 2019; Meekums et al., 2012; Nourizadeh et al., 2020; Nurkkala et al., 2015; Paul et al., 2021; Simos et al., 2019; Stapleton et al., 2016, 2020; Teixeira et al., 2010; Weineland et al., 2012; Yancy et al., 2019; Supp. 3). Twelve (38.7%) studies included only female participants and the proportion of male participants in the remaining studies ranged from 5.5% to 76.1%. The mean baseline BMI ranged from 27.2 to 43.5 kg/m^2^ and the majority of the studies were conducted in the United States (35.5%). The majority of the studies evaluated the effectiveness of CBT (38.7%) on emotional eating and/or weight management using usual care/standard treatment (35.4%) delivered face‐to‐face (64.5%) in individual cum group sessions (54.8%). Interventions lasted from 2 to 48 weeks (Table 1). Emotional eating was commonly measured using the Three‐Factor Eating Questionnaire (TFEQ; emotional eating, uncontrolled eating and cognitive control) versions 18 (TFEQ‐R18; Karlsson et al., 2000; *n* = 13; Cesa et al., 2013; Czepczor‐Bernat et al., 2020; Jarvela‐Reijonen et al., 2018; Kim et al., 2020; Kullgren et al., 2013; Mason et al., 2019; Nourizadeh et al., 2020; Nurkkala et al., 2015; Stapleton et al., 2020; Yancy et al., 2019) and 21(TFEQ‐R21; Cappelleri et al., 2009; *n* = 5; Gade et al., 2013; Hjelmesæth et al., 2019; Kristeller et al., 2014; Teixeira et al., 2010) and the 33‐item Dutch Eating Behaviour Questionnaire (DEBQ; *n* = 5) which also consists of three factors namely emotional eating, external eating and restrained eating. Details of the study characteristics are shown in Table 2.

**TABLE 1:** Summary statistics of study characteristics

Study characteristics	Number of studies
Country
Australia (Stapleton et al., 2016, 2020)	3
Finland (Jarvela‐Reijonen et al., 2018; Nurkkala et al., 2015)	3
Greece (Simos et al., 2019)	1
Iran (Nourizadeh et al., 2020)	1
Italy (Cesa et al., 2013; Manzoni et al., 2016)	4
Korea (Kim et al., 2020)	1
Latvia (Meekums et al., 2012)	1
Norway (Hjelmesæth et al., 2019)	1
Poland (Czepczor‐Bernat et al., 2020)	2
Portugal (Teixeira et al., 2010)	1
Sweden (Weineland et al., 2012)	1
The Netherlands (Paul et al., 2021)	1
United States (Afari et al., 2019; Forman et al., 2013; Gade et al., 2013; Goldbacher et al., 2016; Kristeller et al., 2014; Kullgren et al., 2013; Mason et al., 2019; Yancy et al., 2019)	11
Type of publication	
Conference abstract (Gade et al., 2013)	1
Peer reviewed journal articles	30
Participants with binge eating or emotional eating	
Dysfunctional eating behaviour (Afari et al., 2019; Cesa et al., 2013; Goldbacher et al., 2016; Kristeller et al., 2014; Meekums et al., 2012)	7
Nil	24
Intervention type	
CBT (Cesa et al., 2013; Gade et al., 2013; Goldbacher et al., 2016; Hjelmesæth et al., 2019; Kim et al., 2020; Kristeller et al., 2014; Manzoni et al., 2016; Paul et al., 2021; Stapleton et al., 2016, 2020)	12
CBT + mindfulness (Afari et al., 2019; Czepczor‐Bernat et al., 2020; Forman et al., 2013; Jarvela‐Reijonen et al., 2018; Weineland et al., 2012)	7
Diet and exercise counselling (Mason et al., 2019; Meekums et al., 2012; Nourizadeh et al., 2020; Nurkkala et al., 2015; Stapleton et al., 2020; Teixeira et al., 2010)	7
Financial incentive (Kullgren et al., 2013; Yancy et al., 2019)	3
Mindfulness (Kristeller et al., 2014; Simos et al., 2019)	2
Control condition	
Active control (Kim et al., 2020; Kullgren et al., 2013; Nurkkala et al., 2015; Simos et al., 2019; Stapleton et al., 2016; Stapleton et al., 2020; Yancy et al., 2019)	8
Usual care (Afari et al., 2019; Cesa et al., 2013; Forman et al., 2013; Gade et al., 2013; Goldbacher et al., 2016; Hjelmesæth et al., 2019; Jarvela‐Reijonen et al., 2018; Manzoni et al., 2016; Meekums et al., 2012; Nourizadeh et al., 2020; Paul et al., 2021; Stapleton et al., 2016; Stapleton et al., 2020; Weineland et al., 2012)	17
Wait list (Czepczor‐Bernat et al., 2020; Kristeller et al., 2014; Mason et al., 2019)	6
Interventionist	
Not‐specified (Gade et al., 2013; Hjelmesæth et al., 2019)	2
Trained (Afari et al., 2019; Cesa et al., 2013; Czepczor‐Bernat et al., 2020; Forman et al., 2013; Jarvela‐Reijonen et al., 2018; Kim et al., 2020; Kristeller et al., 2014; Manzoni et al., 2016; Nourizadeh et al., 2020; Paul et al., 2021; Stapleton et al., 2016; Stapleton et al., 2020)	17
Untrained (Goldbacher et al., 2016; Kullgren et al., 2013; Mason et al., 2019; Meekums et al., 2012; Nurkkala et al., 2015; Simos et al., 2019; Stapleton et al., 2020; Teixeira et al., 2010; Weineland et al., 2012; Yancy et al., 2019)	12
Mode of delivery	
Face‐to‐face (Afari et al., 2019; Cesa et al., 2013; Goldbacher et al., 2016; Hjelmesæth et al., 2019; Jarvela‐Reijonen et al., 2018; Kristeller et al., 2014; Manzoni et al., 2016; Mason et al., 2019; Meekums et al., 2012; Nourizadeh et al., 2020; Nurkkala et al., 2015; Paul et al., 2021; Simos et al., 2019; Stapleton et al., 2016; Teixeira et al., 2010; Weineland et al., 2012)	20
Not‐specified (Gade et al., 2013)	1
Self (Forman et al., 2013; Stapleton et al., 2020)	2
Web/app (Czepczor‐Bernat et al., 2020; Jarvela‐Reijonen et al., 2018; Kim et al., 2020; Kullgren et al., 2013; Stapleton et al., 2020; Yancy et al., 2019)	8
Individual/group sessions	
Group (Afari et al., 2019; Goldbacher et al., 2016; Jarvela‐Reijonen et al., 2018; Kullgren et al., 2013; Meekums et al., 2012; Nourizadeh et al., 2020; Stapleton et al., 2016; Teixeira et al., 2010)	8
Individual (Simos et al., 2019; Stapleton et al., 2016; Stapleton et al., 2020; Weineland et al., 2012; Yancy et al., 2019)	5
Individual + group (Cesa et al., 2013; Czepczor‐Bernat et al., 2020; Forman et al., 2013; Hjelmesæth et al., 2019; Jarvela‐Reijonen et al., 2018; Kim et al., 2020; Kristeller et al., 2014; Kullgren et al., 2013; Manzoni et al., 2016; Mason et al., 2019; Nurkkala et al., 2015; Paul et al., 2021)	17
Not‐specified(Gade et al., 2013)	1
Weight measures used	
Calibrated instruments(Cesa et al., 2013; Forman et al., 2013; Goldbacher et al., 2016; Hjelmesæth et al., 2019; Jarvela‐Reijonen et al., 2018; Kristeller et al., 2014; Kullgren et al., 2013; Manzoni et al., 2016; Mason et al., 2019; Meekums et al., 2012; Nourizadeh et al., 2020; Nurkkala et al., 2015; Paul et al., 2021; Simos et al., 2019; Stapleton et al., 2016; Stapleton et al., 2020; Teixeira et al., 2010; Weineland et al., 2012; Yancy et al., 2019)	26
Not‐specified (Afari et al., 2019; Gade et al., 2013)	2
Self‐reported (Czepczor‐Bernat et al., 2020)	2
Emotional eating measures used	25
DEBQ (Kim et al., 2020; Meekums et al., 2012; Paul et al., 2021; Simos et al., 2019)	5
EES (Forman et al., 2013; Goldbacher et al., 2016)	2
Not‐specified (Cesa et al., 2013; Manzoni et al., 2016; Stapleton et al., 2020; Weineland et al., 2012)	6
TFEQ R‐18 (Cesa et al., 2013; Czepczor‐Bernat et al., 2020; Jarvela‐Reijonen et al., 2018; Kim et al., 2020; Kullgren et al., 2013; Mason et al., 2019; Nourizadeh et al., 2020; Nurkkala et al., 2015; Stapleton et al., 2020; Yancy et al., 2019)	13
TFEQ R‐21 (Gade et al., 2013; Hjelmesæth et al., 2019; Kristeller et al., 2014; Teixeira et al., 2010)	5
Follow‐up period on emotional eating	
3‐month post‐intervention (Afari et al., 2019; Czepczor‐Bernat et al., 2020; Kim et al., 2020; Kristeller et al., 2014; Stapleton et al., 2020)	8
6‐month post‐intervention (Afari et al., 2019; Forman et al., 2013; Jarvela‐Reijonen et al., 2018; Yancy et al., 2019)	5
12‐month post‐intervention (Afari et al., 2019; Nurkkala et al., 2015)	2
Follow‐up period on weight change	
3‐month post‐intervention (Afari et al., 2019; Czepczor‐Bernat et al., 2020; Kristeller et al., 2014; Stapleton et al., 2020)	7
6‐month post‐intervention (Forman et al., 2013; Stapleton et al., 2016; Yancy et al., 2019)	4
12‐month post‐intervention(Afari et al., 2019; Cesa et al., 2013; Manzoni et al., 2016; Nurkkala et al., 2015; Teixeira et al., 2010)	8

### 3.2 Risk of bias

35% of the studies scored an overall rating of high RoB (Jarvela‐Reijonen et al., 2018; Kullgren et al., 2013; Manzoni et al., 2016; Meekums et al., 2012; Nourizadeh et al., 2020; Nurkkala et al., 2015; Simos et al., 2019; Stapleton et al., 2020), 39% of studies scored an overall rating of moderate RoB (Afari et al., 2019; Cesa et al., 2013; Gade et al., 2013; Hjelmesæth et al., 2019; Kim et al., 2020; Mason et al., 2019; Paul et al., 2021; Stapleton et al., 2016; Yancy et al., 2019) and 26% of studies scored an overall rating of low RoB (Czepczor‐Bernat et al., 2020; Forman et al., 2013; Goldbacher et al., 2016; Kristeller et al., 2014; Teixeira et al., 2010; Weineland et al., 2012). Interrater agreement was high (kappa = 0.92). The individual domain ratings are detailed in Supp 3.

### 3.3 Intervention effects on emotional eating

**FIGURE 2:**
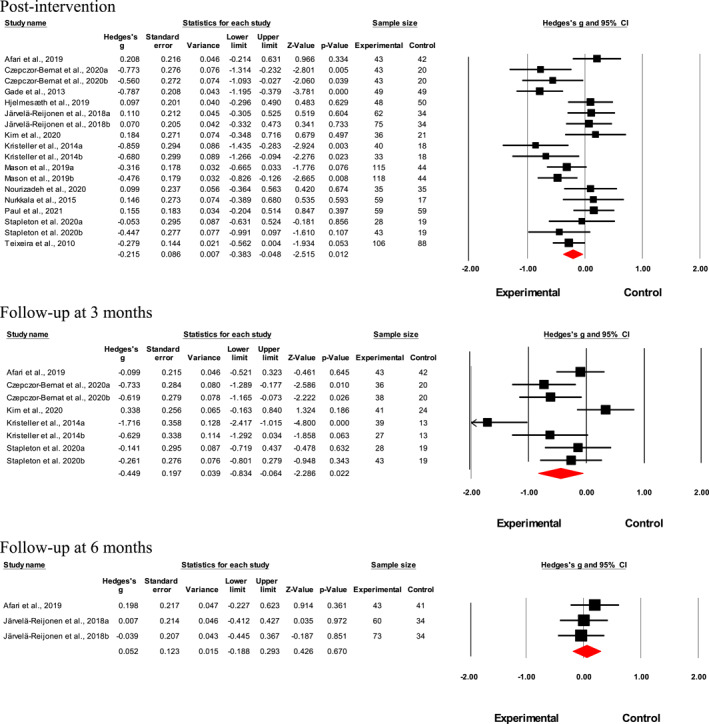
Forest plot of Hedges' *g* in emotional eating for intervention and control groups

Results suggested a small‐to‐medium interventional effect on emotional eating factor post‐intervention (*n* = 18; Hedges' *g* = 0.22; *p* = 0.01, *I* ^2^ = 61.7%; Figure 2). Interventional effects were relatively augmented at 3‐month post‐intervention (*n* = 8; Hedges' *g* = 0.45; *p* = 0.02, *I* ^2^ = 74.3%; Figure 2) but became non‐significant 6‐month post‐intervention (*n* = 3; Hedges' *g* = 0.05; *p* = 0.67, *I* ^2^ < 0%; Figure 2). Meta‐analysis was not performed for results at 12‐month post‐intervention as only two studies were available, and it would not have provided sufficient power for a reliable conclusion. No publication bias was found for the intervention effects on emotional eating factor directly post‐intervention as shown in the funnel plot (Supp. 4) and Egger's test (*t* = 0.59; 95% CI [−4.92, 2.77], *p* = 0.28).

Meta‐regression results showed that age, the proportion of male participants, baseline BMI, attrition rate and intervention length did not significantly moderate the heterogeneity between study effect sizes (Table 3). Subgroup analyses showed that the interventional effect was larger in studies that compared the intervention group against a waitlist control group (*n* = 6; Hedges' *g* = 0.54; *Q* = 13.1, *p* = 0=.001) (Supp. 6).

**TABLE 2:** Study characteristics

Author, year	Country	Type of publication	N	Mean age	% Male	Baseline BMI	Participants characteristics	Intervention	Intervention type
Afari et al., 2019	US	IPR	88	57.3	76.1	37.2	Self‐identified as having problems with ‘stress‐related eating’	ACT	ACT
Cesa et al., 2013	Italy	IPR	90	32.6	0	40.3	Binge‐eating disorder	VR‐enhanced CBT	CBT + VR
	Italy	IPR	90	32.6	0	40.3	Binge‐eating disorder	CBT	CBT
Czepczor‐Bernat et al., 2020	Poland	IPR	129	32.28	0	30.5	No	TCA, MET, and CPBID	ACT
Czepczor‐Bernat et al., 2020	Poland	IPR	0	32.3	0	30.5	No	EA, MET, and CPBID	ACT
Forman et al., 2013	US	IPR	128	45.7	NS	34.1	No	Acceptance‐based behavioural treatment	ACT
Gade et al., 2013	US	Conference abstract	98	43.0	30.6	43.5	No	CBT	CBT
Goldbacher et al., 2016	US	IPR	79	45.6	5	36.2	Top tertile on any of the three subscales of the emotional eating scale	Enhanced behavioural treatment (EBT) incorporating skills for managing emotions and emotional eating	CBT
Hjelmesæth et al., 2019	Norway	IPR	98	42.4	30	43.5	No	CBT	CBT
Järvelä‐Reijonen et al., 2018	Finland	IPR	219	49.5	15	31.3	No	ACT	ACT
Järvelä‐Reijonen et al., 2018	Finland	IPR	0	49.5	15	31.3	No	ACT	ACT
Kim et al., 2020	Korea	IPR	70	21.8	0	28	No	CBT + Noom Coach app (personalised health coaching)	CBT + self‐regulation
Kristeller et al., 2014	US	IPR	150	46.6	12	40.3	Binge‐eating disorder	Mindfulness‐based eating awareness training	Mindfulness
Kristeller et al., 2014	US	IPR	0	46.6	12	40.3	Binge‐eating disorder	Psychoeducational/cognitive–behavioural intervention	CBT
Kullgren et al., 2013	US	IPR	105	45.3	11	4.6	No	$100 per person per month for meeting or exceeding weight‐loss goals	Financial incentive
Kullgren et al., 2013	US	IPR	0	45.3	11	4.6	No	$500 per month split among participants within groups of 5 who met or exceeded weight‐loss goals	Financial incentive
Manzoni et al., 2016	Italy	IPR	158	35.6	0	42.2	No	VR‐enhanced CBT	CBT + VR
Manzoni et al., 2016	Italy	IPR	0	35.6	0	42.2	No	CBT	CBT
Mason et al., 2019	US	IPR	439	57.8	0	30.7	No	Aerobic exercise (moderate‐to‐vigorous intensity aerobic exercise for 225 min/week) + activity logging	Exercise + activity logging
Mason et al., 2019	US	IPR	0	57.8	0	30.7	No	Calorie‐reduced diet (dietary weight loss with a 10% weight‐loss goal) + counselling	Diet + counselling
Meekums et al., 2012	Latvia	IPR	158	40	0	NS	Self‐reported emotional eating	Dance movement therapy + counselling	Exercise + counselling
Nourizadeh et al., 2020	Iran	IPR	70	28.5	0	30.3	No	Motivational interviewing	Self‐regulation counselling
Nurkkala et al., 2015	Finland	IPR	76	45	27.6	35.6	No	Weight maintenance counselling three times by a nutritionist and 11 times by a qualified nurse	Self‐regulation counselling
Paul et al., 2021	The Netherlands	IPR	130	41.7	26	43	No	CBT	CBT
Simos et al., 2019	Greece	IPR	49	53.5	20.4	31.3	No	Pythagorean self‐awareness intervention & personalised Mediterranean low‐calorie diet	Mindfulness
Stapleton et al., 2016	Australia	IPR	83	49.3	10.5	33.7	No	EFT	CBT + somatic stimulation
Stapleton et al., 2020	Australia	IPR	343	47.4	5.5	36.9	No	Portion perfection for bariatric patients (PPBP)	Self‐regulation counselling
Stapleton et al., 2020	Australia	IPR	240	47.35	5.5	37.3	No	PPBP + EFT	CBT + somatic stimulation
Teixeira et al., 2010	Portugal	IPR	225	37.6	0	31.3	No	Intervention designed to promote autonomous self‐regulation of body weight	Self‐regulation counselling
Weineland et al., 2012	Sweden	IPR	39	43.1	10.3	27.2	No	ACT	ACT
Yancy et al., 2019	US	IPR	258	48.0	12.8	32.1	No	Escalating lottery‐based incentive tied to daily self‐weighing for weight loss maintenance	Financial incentive

Abbreviations: ACT, Acceptance and commitment therapy; BMI, body mass index; CBT, Cognitive behaviour therapy; CPBID, Cash's prevention of body image disturbances; EFT, Emotional freedom technique (EFT) which combines aspects of exposure and cognitive therapy with somatic stimulation through acupressure points; IPR, internationally peer‐reviewed; MET, mindfulness‐based eating training; TCA, theoretically consistent approach based on Emotional Schema Therapy to increased level of acceptance of emotions; VR, Virtual reality.

### 3.4 Intervention effects on uncontrolled eating/external eating

**FIGURE 3:**
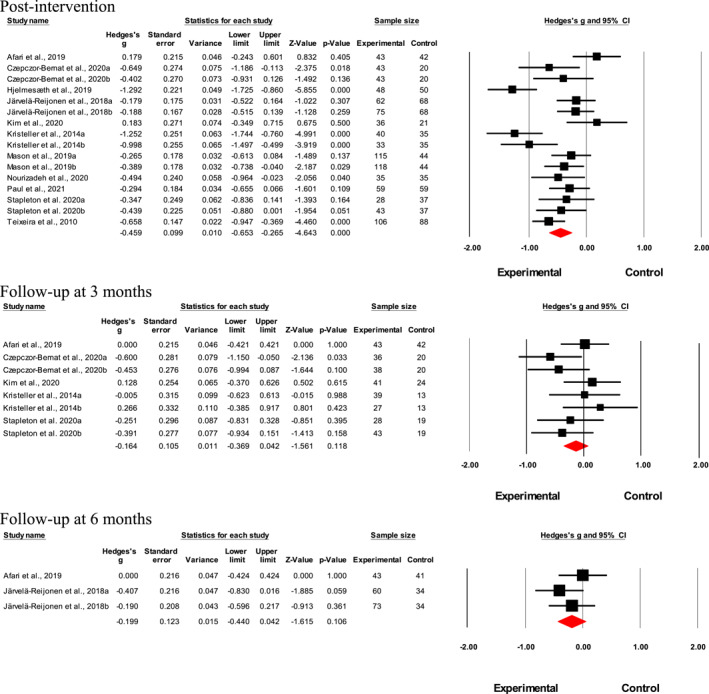
Forest plot of Hedges' g in uncontrolled eating for intervention and control groups

Results suggested a small‐to‐medium interventional effect on uncontrolled eating post‐intervention (*n* = 16; Hedges' *g* = 0.46; *p* < 0.001, *I* ^2^ = 71.6%; Figure 3). Although not statistically significant, interventional effects declined 3‐month post‐intervention (*n* = 8; Hedges' *g* = 0.16; *p* = 0.12, *I* ^2^ = 13.6%; Figure 3) and at 6‐month post‐intervention (*n* = 3; Hedges' *g* = 0.20; *p* = 0.11, *I* ^2^ < 0%; Figure 3). Meta‐analysis was not performed for results at 12‐month‐post intervention as only two studies were available, and it would not have provided sufficient power for a reliable conclusion. No publication bias was found for the intervention effects on uncontrolled eating factor directly post‐intervention according to the funnel plot (Supp. 5) and Egger's test (*t* = 0.69; 95% CI [‐6.93, 3.56]; *p* = 0.50).

Meta‐regression results showed that baseline BMI was a significant moderator of the heterogeneity in study effects (Table 3). Findings from the subgroup analyses showed that groups that underwent CBT, diet and/or exercise and mindfulness had a larger intervention effect than CBT cum mindfulness (*n* = 5; Hedges' *g* = 0.57; *Q* = 14.5, *p* = 0.002) (Table 4).

**TABLE 3:** Random effects meta‐regression models of emotional eating, uncontrolled eating, cognitive restraint, weight and BMI by various covariates

Covariates	*n*	*β*	SE	95% CI	*Z*‐score	*p*‐Value	*I* ^2^(%)
Age							
Emotional eating							
Uncontrolled eating	18	−0.00	0.01	−0.02, 0.02	−0.15	0.88	63.9
Cognitive restraint	16	0.00	0.01	−0.02, 0.02	0.33	0.74	72.5
BMI	18	0.01	0.01	−0.03, 0.01	−0.79	0.43	76.3
Weight	11	0.03	0.03	−0.02, 0.08	1.32	0.19	85.9
% Male participants							
Emotional eating	18	0.01	0.00	−0.00, 0.02	1.58	0.11	58.0
Uncontrolled eating	16	0.00	0.01	−0.01, 0.01	0.81	0.42	72.0
Cognitive restraint	18	‐0.01	0.01	−0.02, 0.01	‐0.91	0.36	75.7
BMI	11	‐0.00	0.02	−0.04, 0.04	‐0.01	0.99	86.3
Baseline BMI							
Emotional eating	18	‐0.01	0.02	−0.04, 0.03	‐0.40	0.69	64.0
Uncontrolled eating	16	‐0.04	0.02	−0.08, 0.00	−2.21*	0.03	67.1
Cognitive restraint	18	0.01	0.1	−0.01, 0.03	0.76	0.45	76.5
BMI	11	‐0.08	0.05	−0.17, 0.02	−1.54	0.13	84.9
Attrition rate							
Emotional eating	18	‐0.00	0.00	−0.01, 0.01	−0.80	0.42	62.7
Uncontrolled eating	16	‐0.00	0.00	−0.01, 0.01	−0.86	0.39	71.8
Cognitive restraint	18	0.00	0.01	−0.01, 0.02	0.40	0.69	77.1
BMI	11	‐0.00	0.01	−0.03, 0.02	−0.17	0.86	86.8
Intervention length							
Emotional eating	18	‐0.00	0.01	−0.01, 0.01	−0.05	0.62	62.2
Uncontrolled eating	16	0.01	0.01	−0.01, 0.01	−0.22	0.83	73.2
Cognitive restraint	18	0.01	0.01	−0.01, 0.02	1.06	0.29	75.8
BMI	11	‐0.03	0.03	−0.08, 0.03	−0.95	0.34	86.0

Abbreviations: BMI, body mass index; CI, confidence interval; *n*, number of studies; SE, Standard error.

*p‐value ≤ 0.05; *p‐value ≤ 0.01; *p‐value ≤ 0.001.

### 3.5 Intervention effects on cognitive restraint/restrained eating

**FIGURE 4:**
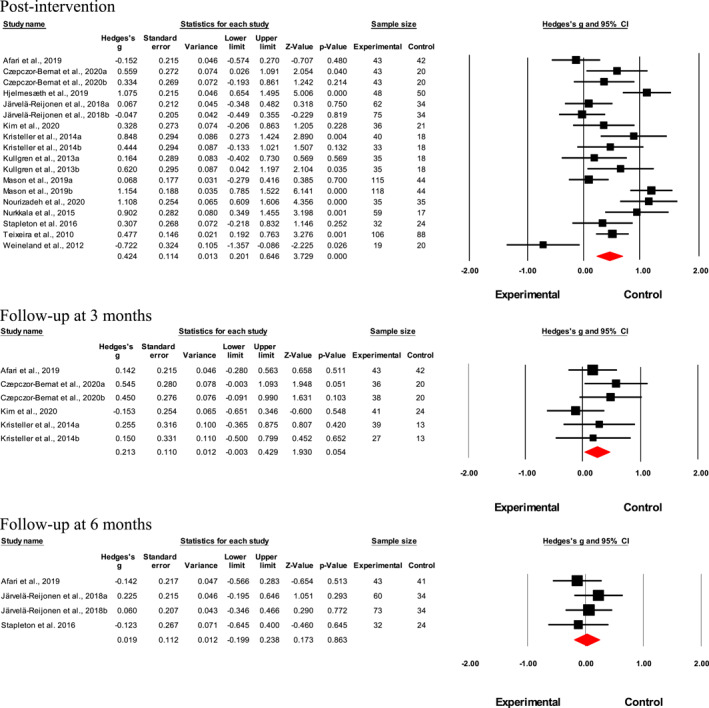
Forest plot of Hedges' *g* in cognitive restraint for intervention and control groups Post‐intervention

Results suggested a small‐to‐medium interventional effect on the cognitive restraint factor directly post‐intervention (*n* = 18; Hedges' *g* = 0.42; *p* < 0.001, *I* ^2^ = 75.8%; Figure 4). Although not statistically significant, interventional effects declined 3‐month post‐intervention (*n* = 6; Hedges' *g* = 0.21; *p* = 0.054, *I* ^2^ < 0%; Figure 4) and at 6‐month post‐intervention (*n* = 4; Hedges' *g* = 0.02; *p* = 0.86, *I* ^2^ < 0%; Figures 4, 6). Meta‐analysis was not performed for results at 12‐month post‐intervention as only two studies were available, and it would not have provided sufficient power for a reliable conclusion. No publication bias was found for the intervention effects on Cognitive restraint factor directly post‐intervention according to the funnel plot (Supp. 6) and Egger's test (*t* = 0.17; 95% CI [−5.06; 4.29]; *p* = 0.86).

Meta‐regression results showed that none of the covariates added into the random effects models was significantly moderators of the heterogeneity in study effects (Table 3, 4). Subgroup analyses showed that the interventional effect was larger in studies that used CBT, diet and/or exercise and mindfulness than CBT cum mindfulness (Table 4). However, only one study used a purely mindfulness intervention.

**TABLE 4:** Subgroup analyses on emotional eating, uncontrolled eating, cognitive restraint, weight and BMI

Outcomes	Subgroups	*n*	*g*	*Q*‐value	*p*‐value
Emotional eating	Region			5.23	0.16
	Eastern Mediterranean (Nourizadeh et al., 2020)	1	0.10		
	Europe (Czepczor‐Bernat et al., 2020; Hjelmesæth et al., 2019; Jarvela‐Reijonen et al., 2018; Nurkkala et al., 2015; Paul et al., 2021; Teixeira et al., 2010)	8	0.11		
	United States (Afari et al., 2019; Gade et al., 2013; Kristeller et al., 2014; Mason et al., 2019)	6	0.46		
	Western Pacific (Kim et al., 2020; Stapleton et al., 2020)	3	0.10		
	Dysfunctional eating			0.40	0.53
	Yes (Afari et al., 2019; Kristeller et al., 2014; Meekums et al., 2012)	3	0.42		
	No (Czepczor‐Bernat et al., 2020; Gade et al., 2013; Hjelmesæth et al., 2019; Jarvela‐Reijonen et al., 2018; Kim et al., 2020; Mason et al., 2019; Nourizadeh et al., 2020; Nurkkala et al., 2015; Paul et al., 2021; Stapleton et al., 2020; Teixeira et al., 2010)	15	0.19		
	Intervention type			4.7	0.19
	CBT (Gade et al., 2013; Hjelmesæth et al., 2019; Kim et al., 2020; Kristeller et al., 2014; Paul et al., 2021; Stapleton et al., 2020)	6	0.23		
	CBT + mindfulness (Afari et al., 2019; Czepczor‐Bernat et al., 2020; Jarvela‐Reijonen et al., 2018)	5	0.16		
	Diet &/exercise (Mason et al., 2019; Meekums et al., 2012; Nourizadeh et al., 2020; Nurkkala et al., 2015; Stapleton et al., 2020; Teixeira et al., 2010)	6	0.21		
	Mindfulness (Kristeller et al., 2014)	1	0.86		
	Control condition			13.1***	0.001
	Active (Kim et al., 2020; Nurkkala et al., 2015; Teixeira et al., 2010)	3	0.05		
	Usual care (Afari et al., 2019; Gade et al., 2013; Hjelmesæth et al., 2019; Jarvela‐Reijonen et al., 2018; Meekums et al., 2012; Nourizadeh et al., 2020; Paul et al., 2021; Stapleton et al., 2020)	9	0.05		
	Waitlist (Czepczor‐Bernat et al., 2020; Kristeller et al., 2014; Mason et al., 2019)	6	0.54		
Uncontrolled eating	Region			1.77	0.62
	Eastern Mediterranean (Nourizadeh et al., 2020)	1	0.49		
	Europe (Czepczor‐Bernat et al., 2020; Hjelmesæth et al., 2019; Jarvela‐Reijonen et al., 2018; Paul et al., 2021; Teixeira et al., 2010)	7	0.51		
	United States (Afari et al., 2019; Kristeller et al., 2014; Mason et al., 2019)	5	0.53		
	Western Pacific (Kim et al., 2020; Stapleton et al., 2020)	3	0.22		
	Dysfunctional eating			0.32	0.57
	Yes (Afari et al., 2019; Kristeller et al., 2014)	3	0.68		
	No (Czepczor‐Bernat et al., 2020; Hjelmesæth et al., 2019; Jarvela‐Reijonen et al., 2018; Kim et al., 2020; Mason et al., 2019; Nourizadeh et al., 2020; Paul et al., 2021; Stapleton et al., 2020; Teixeira et al., 2010)	13	0.42		
	Intervention type			14.5**	0.002
	CBT (Hjelmesæth et al., 2019; Kim et al., 2020; Kristeller et al., 2014; Paul et al., 2021; Stapleton et al., 2020)	5	0.57		
	CBT + mindfulness (Afari et al., 2019; Czepczor‐Bernat et al., 2020; Jarvela‐Reijonen et al., 2018)	5	0.21		
	Diet &/exercise (Mason et al., 2019; Nourizadeh et al., 2020; Stapleton et al., 2020; Teixeira et al., 2010)	5	0.46		
	Mindfulness (Kristeller et al., 2014)	1	1.25		
	Control condition			1.77	0.41
	Active (Kim et al., 2020; Teixeira et al., 2010)	2	0.27		
	Usual care (Afari et al., 2019; Hjelmesæth et al., 2019; Jarvela‐Reijonen et al., 2018; Nourizadeh et al., 2020; Paul et al., 2021; Stapleton et al., 2020)	8	0.37		
	Waitlist (Czepczor‐Bernat et al., 2020; Kristeller et al., 2014; Mason et al., 2019)	5	0.64		
Cognitive restraint	Region			7.42	0.06
	Eastern Mediterranean (Nourizadeh et al., 2020)	1	1.11		
	Europe (Czepczor‐Bernat et al., 2020; Hjelmesæth et al., 2019; Jarvela‐Reijonen et al., 2018; Nurkkala et al., 2015; Paul et al., 2021; Teixeira et al., 2010)	8	0.35		
	United States (Afari et al., 2019; Kristeller et al., 2014; Mason et al., 2019)	7	0.45		
	Western Pacific (Kim et al., 2020; Stapleton et al., 2020)	2	0.32		
	Dysfunctional eating			0.06	0.80
	Yes (Afari et al., 2019; Kristeller et al., 2014)	3	0.35		
	No (Czepczor‐Bernat et al., 2020; Hjelmesæth et al., 2019; Jarvela‐Reijonen et al., 2018; Kim et al., 2020; Kullgren et al., 2013; Mason et al., 2019; Nourizadeh et al., 2020; Nurkkala et al., 2015; Stapleton et al., 2020; Teixeira et al., 2010; Weineland et al., 2012)	15	0.44		
	Intervention type			11.6*	0.02
	CBT (Hjelmesæth et al., 2019; Kim et al., 2020; Kristeller et al., 2014; Stapleton et al., 2016)	4	0.56		
	CBT + mindfulness (Afari et al., 2019; Czepczor‐Bernat et al., 2020; Jarvela‐Reijonen et al., 2018; Weineland et al., 2012)	6	0.02		
	Diet &/exercise (Mason et al., 2019; Nourizadeh et al., 2020; Nurkkala et al., 2015; Teixeira et al., 2010)	5	0.72		
	Financial incentive (Kullgren et al., 2013)	2	0.39		
	Mindfulness (Kristeller et al., 2014)	1	0.85		
	Control condition			1	0.61
	Active (Kim et al., 2020; Kullgren et al., 2013; Nurkkala et al., 2015; Stapleton et al., 2016; Teixeira et al., 2010)	6	0.47		
	Usual care(Afari et al., 2019; Hjelmesæth et al., 2019; Jarvela‐Reijonen et al., 2018; Nourizadeh et al., 2020; Weineland et al., 2012)	6	0.23		
	Waitlist (Czepczor‐Bernat et al., 2020; Kristeller et al., 2014; Nourizadeh et al., 2020)	6	0.57		
BMI	Region			2.63	0.27
	Europe (Cesa et al., 2013; Hjelmesæth et al., 2019; Nurkkala et al., 2015; Simos et al., 2019)	5	0.03		
	United States (Kristeller et al., 2014)	2	0.16		
	Western Pacific (Kim et al., 2020; Stapleton et al., 2016, 2020)	4	0.19		
	Dysfunctional eating	4	0.02	0.04	0.85
	Yes(Cesa et al., 2013; Kristeller et al., 2014)	7			
	No (Hjelmesæth et al., 2019; Kim et al., 2020; Nurkkala et al., 2015; Simos et al., 2019; Stapleton et al., 2016, 2020)	7	0.05		
	Intervention type			2.93	0.23
	CBT(Cesa et al., 2013; Hjelmesæth et al., 2019; Kim et al., 2020; Kristeller et al., 2014; Stapleton et al., 2016, 2020)	7	0.29		
	Diet &/exercise(Nurkkala et al., 2015; Stapleton et al., 2020)	2	0.57		
	Mindfulness(Kristeller et al., 2014)	2	1.26		
	Control condition			7.62*	0.02
	Active (Kim et al., 2020; Nurkkala et al., 2015; Simos et al., 2019; Stapleton et al., 2016)	4	0.03		
	Usual care (Cesa et al., 2013; Hjelmesæth et al., 2019; Stapleton et al., 2016, 2020)	5	0.46		
	Waitlist (Kristeller et al., 2014)	2	0.16		

Abbreviations: BMI, body mass index; CBT, cognitive behavioural therapy; CI, confidence interval; *n*, number of studies; ***g***, Hedges' *g*; SE, standard error.

*p‐value ≤ 0.05; **p‐value ≤ 0.01; ***p‐value ≤ 0.001.

### 3.6 Intervention effects on binge eating

**FIGURE 5:**
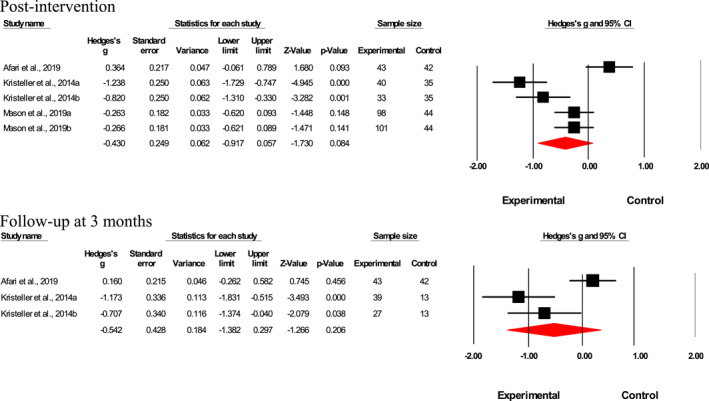
Forest plot of Hedges' *g* in binge‐eating behaviour for intervention and control groups post‐intervention

Results suggested no significant interventional effect on binge‐eating behaviour post‐intervention (*n* = 5; Hedges' *g* = 0.43; *p* = 0.08, *I* ^2^ = 85.4%) and 3‐month post‐intervention (*n* = 3; Hedges' *g* = 0.54; *p* = 0.21, *I* ^2^ = 84.3%; Figure 5). Meta‐analysis was not performed for results at 6‐ and 12‐month post‐intervention as only one study was available, and it would not have provided sufficient power for a reliable conclusion. As there were only five included studies that reported changes in binge‐eating behaviour, a meta‐regression and subgroup analysis was not performed as it would have been underpowered.

### 3.7 Intervention effects on BMI

**FIGURE 6:**
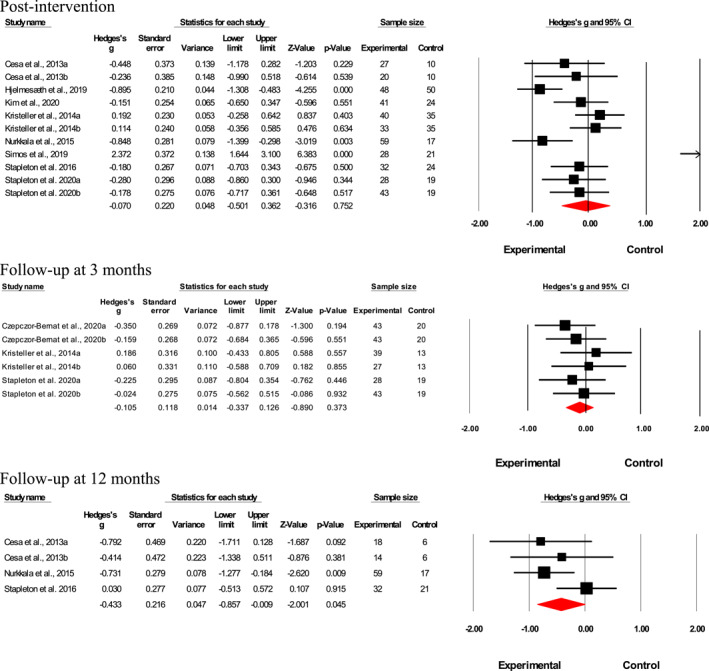
Forest plot of Hedges' *g* in body mass index for intervention and control groups. Directly post‐intervention

Results suggested no significant interventional effect on weight measured in BMI directly post‐intervention (*n* = 11; Hedges' *g* = 0.07; *p* = 0.75, *I* ^2^ = 85.5%) and 3‐month post‐intervention (*n* = 6; Hedges' *g* = 0.11; *p* = 0.37, *I* ^2^ < 0%; Figure 6). However, a small‐to‐medium effect of such interventions on weight loss was found 12‐month post‐intervention (*n* = 4; Hedges' *g* = 0.43; *p* < 0.05, *I* ^2^ = 33.4%; Figure 6). Meta‐analysis was not performed for results at 6‐month post‐intervention as only one study was available and it would not have provided sufficient power for a reliable conclusion. According to the funnel plot and Egger's test (*t* = 1.3; *p* = 0.23), no publication bias was found for the intervention effects on uncontrolled eating factor directly post‐intervention.

Meta‐regression results showed that baseline BMI was a significant moderator of the heterogeneity in study effects (Table 3). Subgroup analyses showed that the interventional effect was larger in studies that compared the intervention group against a usual care group (Table 4).

### 3.8 Intervention effects on weight (kg)

**FIGURE 7:**
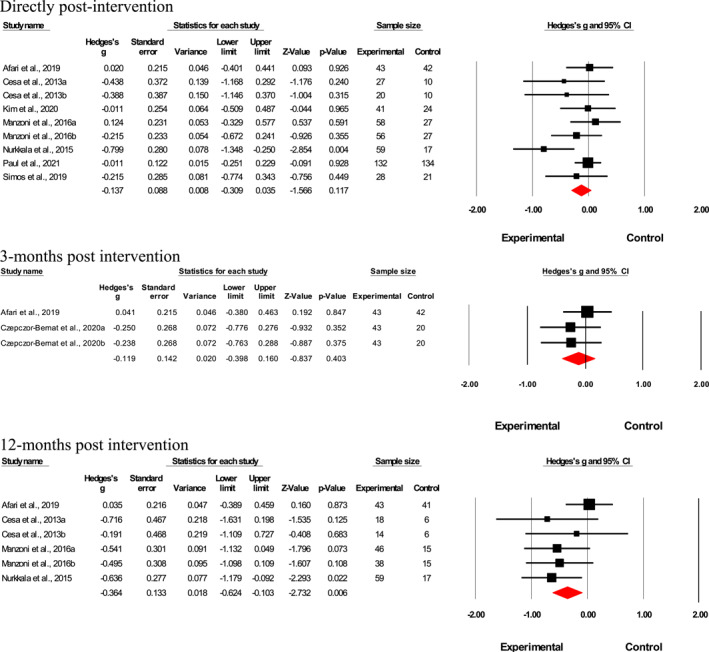
Forest plot of Hedges' *g* in weight (kg) for intervention and control groups

Results suggested no significant effect of emotional eating interventions on the participants' weight measured in kg directly post‐intervention (*n* = 9; Hedges' *g* = 0.14; *p* = 0.12, *I* ^2^ = 19.1%) and 3‐month post‐intervention (*n* = 3; Hedges' *g* = 0.12; *p* = 0.40, *I* ^2^ < 0%; Figure 7). However, a small‐to‐medium effect of such interventions on weight loss was found 12‐month post‐intervention (*n* = 6; Hedges' *g* = 0.36; *p* < 0.01, *I* ^2^ < 10.4%; Figure 7). Meta‐analysis was not performed for results at 6‐month‐post intervention as only one study was available and it would not have provided sufficient power for a reliable conclusion. As there were only nine included studies that reported changes in weight (kg), a meta‐regression and subgroup analysis was not performed as it would have been underpowered.

## 4 DISCUSSION

### 4.1 Overall findings

In summary, we found evidence to support the effectiveness of weight‐loss interventions such as CBT, diet and exercise and mindfulness on improving emotional eating. It is noteworthy that weight loss could be a by‐product instead of the aim of certain interventions such as CBT, which prioritises the aim of resolving a psychological issue rather than weight loss. We found an overall small‐to‐medium post‐interventional effect size on improving emotional eating, uncontrolled eating/external eating and cognitive restraint/restrained eating, regardless of intervention length (2–48 weeks). Specifically, purely mindfulness‐based interventions showed a higher interventional effect size over a combination of CBT and mindfulness, CBT and diet and/or exercise. Small‐to‐medium interventional effect size on BMI and weight was only observed 12‐month post‐intervention. Interestingly, interventional effects were augmented 3‐month post‐intervention but only for emotional eating. The significant effect size became non‐significant for uncontrolled eating/external eating and cognitive restraint/restrained eating. Interventional effects on emotional eating, uncontrolled eating/external eating and cognitive restraint/restrained eating became non‐significant 6‐month post‐intervention. We were surprised to find that subgroup differences in intervention type was only found for uncontrolled eating and cognitive restraint but not emotional eating, weight and BMI. On the other hand, heterogeneity in emotional eating and BMI outcomes were moderated by baseline BMI and differed between studies that used active, usual care or waitlist control group.

Our findings suggest that interventions targeted at reducing emotional eating could have direct effects on uncontrolled eating and cognitive restraint but delayed effects on emotional eating, weight and BMI. Given that controlled eating and cognitive restraint are dynamic self‐regulation skill that takes time to hone (Miller et al., 2020), we speculate that the interventions influence emotional eating, weight and BMI indirectly through higher self‐regulation (e.g., lower uncontrolled eating and higher cognitive restraint). Self‐regulation refers to the ability to control and monitor one's thoughts, emotions and behaviours (Miller et al., 2020). However, this must be supported by more rigorous evidence.

The finding that interventional effects on emotional eating, uncontrolled eating/external eating and cognitive restraint/restrained eating were unsustainable beyond 6‐month post‐intervention led us to two speculations. Firstly, the termination of weight‐loss interventions could have decreased the participants' motivation to continue with their weight‐loss efforts, especially when they felt discouraged from experiencing a weight‐loss plateau or a relapse of disinhibited eating habits (Montesi et al., 2016). However, this does not explain the pooled effect of significant weight loss 1‐year post‐intervention, assuming that we have accounted for all the covariates. Therefore, our second speculation was that the interventional effects were sustained but masked by an increase in self‐regulation, where participants no longer felt the exertion of cognitive restraint over uncontrolled eating (Chew et al., 2019). In other words, participants have successfully improved their eating habits such they did not feel the cognitive effort to self‐regulate their eating behaviours. This is supported by a systematic review where weight management was found to be mediated by intrinsic motivation, confidence, self‐regulation skills and flexible cognitive restraint (Teixeira et al., 2015). However, this speculation has to be further supported by more rigorous RCTs that account for the mentioned variables.

Overall, our findings suggest that interventions such as CBT, diet and exercise and mindfulness could result in a significant weight loss but only after 1‐year post‐intervention. This delay could be due to the time needed to hone self‐regulation skills through the consistent practice of self‐regulation for weight loss to be significant. One possible mechanism underlying this relationship could be the gradual improvement in self‐regulation, where one trains the cognitive restraint over uncontrolled eating (Chew et al., 2021; Johnson et al., 2012). This is supported by previous studies where self‐regulation was found to be a mediator of the relationship between mood, stress and emotional eating (Annesi, 2019; Ling & Zahry, 2021). However, our speculation has to be further supported by more rigorous research methods such as moderation analyses and RCTs. Comparison of interventional effects could also be done through a network meta‐analysis. To our best knowledge, there is currently no network meta‐analysis that compares the effectiveness between CBT, mindfulness and lifestyle modifications on weight loss.

Ironically, there was no significant interventional effect on binge eating although its phenotypical characteristic of eating a large amount of food at a sitting is somewhat similar to uncontrolled eating. This could be due to the vast difference in their psychopathological nature whereas that of binge eating is commonly due to a more complex underlying psychological burden. People living with binge‐eating disorders often have a reduced reward sensitivity that could compromise the long‐term effectiveness of such interventions (Schag et al., 2021). On the other hand, a lack of significant findings could have been due to an underpowered meta‐analysis and high heterogeneity as compared with the one on uncontrolled eating (*N* = 514 vs. 1539 directly post‐intervention and 177 vs. 356 3‐month post‐intervention; *I* ^2^ = 85.4% vs. 71.6% directly post‐intervention and 84.3% vs. 13.6% 3‐month post‐intervention). The discrepancy in results could have also been due to the use of different scales as uncontrolled eating was mostly measured using the TEFQ R19 9‐item subscale or DEBQ 10‐item subscale while that of binge eating was measured using the 16‐item Binge Eating Scale (BES). On the other hand, binge eating could represent a more severe form of uncontrolled eating along a continuum, so severe that it is associated with impaired executive function (Prunell‐Castañé et al., 2021). In this case, our findings could have suggested minimal interventional effects on binge‐eating phenotype due to its severity. However, more rigorous studies comparing the interventional effects on populations with binge eating and uncontrolled eating phenotypes should be conducted to support this point.

Although subgroup analyses are often underpowered to make any true judgements about subgroup differences (Burke et al., 2015), we highlighted a noteworthy finding that mindfulness‐based interventions seemed to have a greater effect on all emotional eating, uncontrolled eating, cognitive restraint and BMI than the other interventions. Interestingly, interventions with a mixture of CBT and mindfulness seemed to have a counter‐productive effect as shown by a lower effect size. This contradicts a study where greater weight loss was reported in participants who underwent a combination of a 6‐week mindfulness‐based stress reduction and cognitive behavioural stress‐eating intervention as compared to those who received either. However, this was specific to participants who reported stress‐eating and was underpowered given the small sample size of 53 overweight participants made up of 98% females split into three experimental groups. In our study, seven studies included participants with stress‐eating or binge‐eating habits of which six reported significantly lower emotional eating tendencies in participants who received ACT (Afari et al., 2019; Cesa et al., 2013; Kristeller et al., 2014; Meekums et al., 2012), CBT, mindfulness‐based eating awareness training and dance movement than those who received standard behavioural treatment therapy or no treatment. This coincides with a prior systematic review that reported the effectiveness of third wave CBT on emotional eating, dietary restraint, disinhibition and hunger (Lawlor et al., 2020). One study found no differences in emotional eating between the group that received CBT and standard treatment (Goldbacher et al., 2016). This could be due to the interventionist being untrained in CBT, resulting in lower treatment fidelity and treatment effect (Kechter et al., 2019). Further studies could examine the active components of the psycho‐behavioural interventions and the underlying mechanism by which these components influence emotional eating and weight.

### 4.2 Limitations

There were several limitations to this study. First, we did not include relevant studies written in non‐English language although we tried to do so with papers written in Chinese language. This could have omitted relevant studies from other countries and cultures, reducing the generalisability of our findings. Secondly, our findings were limited by the high RoB in 35% of the included studies. Thirdly, findings derived from subgroup analyses were prone to false positives and false negatives due to multiplicity (where multiple subgroups are tested; Barraclough & Govindan, 2010) and insufficient power, respectively (Burke et al., 2015). Moderator analyses were also not conducted for moderators evaluated in the meta‐regression to support our speculated indirect interventional effects on emotional eating and weight through uncontrolled eating and cognitive restraint. Lastly, findings may be limited generalizability because most of the studies were conducted in United States (only one Korean study conducted in Asia) and were mostly on female participants (96.8% female majority; 38.7% solely on females). Future studies should consider the difference in intervention effects on other subgroups such as ethnicity and socio‐economic statuses. It is also noteworthy that studies were not consistent in reporting the use of standard protocols for their psychotherapeutic interventions such as CBT.

## 5 CONCLUSION

This comprehensive systematic review and meta‐analyses showed that mindfulness‐based interventions, CBT, and diet and exercise have a small‐to‐medium effect on improving emotional eating and weight loss along a temporal trajectory. A proposed mechanism is that such interventions improve cognitive restraint over uncontrolled eating post‐intervention, of which effects are translated into reduced emotional eating habits that are only observed 3‐month post‐intervention and weight loss 1‐year post‐intervention. However, this speculation is to be examined using more rigorous methodologies such as RCTs and longitudinal studies. There was insufficient evidence on the effects of financial incentives due to the limited outcomes of interest reported in the relevant studies, limiting the inclusion of sufficient studies on financial incentives into the meta‐analyses. Future studies could consider evaluating the construct of self‐regulation and habits to ascertain our speculation. Larger studies with rigorous methodologies are warranted to elucidate the active components of the examined interventions to streamline weight‐loss programmes for enhanced effectiveness and efficiency.

## CONFLICT OF INTEREST

The authors declare no conflict of interest.

## AUTHOR CONTRIBUTIONS

**Han Shi Jocelyn Chew**: Conceptualization, methodology, software, validation, formal analysis, investigation, data curation, writing ‐ original draft, writing – review and editing, visualization. **Siew Tiang Lau**: Conceptualization, software, validation, formal analysis, writing – review and editing. **Ying Lau**: Conceptualization, methodology, software, formal analysis, writing – review and editing.

## Supporting information

Supporting Information S1

## Data Availability

Data sharing is not applicable to this article as no new data were created or analyzed in this study.
